# Chronic Exposure to Niclosamide Disrupts Structure and Metabolism of Digestive Glands and Foot in *Cipangopaludina cathayensis*

**DOI:** 10.3390/biology15010102

**Published:** 2026-01-04

**Authors:** Yanan Zhang, Yizhen Liu, Qiying Cai, Jun Ye, Tao Wang, Sheng Xu, Gang Ge

**Affiliations:** 1 School of Life Science, Key Laboratory of Poyang Lake Environment and Resource Utilization, Ministry of Education, Nanchang University, Nanchang 330031, China; 2 Jiangxi Provincial Key Laboratory of Poyang Basin Ecological Hydrological Monitoring, Nanchang 330031, China

**Keywords:** Gastropoda, histopathology, mesocosm, nutritional coupling, environmental safety

## Abstract

Niclosamide has been the primary molluscicide for schistosomiasis control for over 50 years, but its chronic effects on inter-organ interactions in non-target mollusks remain poorly understood. *Cipangopaludina cathayensis*, a dominant species in East Asian schistosomiasis-endemic regions, was chronically exposed to environmentally relevant concentrations of niclosamide to assess its toxic effects. Digestive glands accumulated more niclosamide than the foot tissues. Prolonged exposure was associated with metabolic impairment of the digestive glands, characterized by tubular atrophy, inflammatory reactions, and depletion of nutrient components. Foot tissues exhibited epithelial lesions and muscle fiber atrophy. Alterations in foot structure were associated with changes in digestive gland nutrient status. Niclosamide exposure may weaken the metabolic coupling between these organs, thereby impairing locomotor function. At the population level, persistent niclosamide exposure may destabilize mollusk trophic-level populations, ultimately leading to ecological consequences. Our findings demonstrate the toxicological risks of niclosamide to freshwater mollusks.

## Simple Summary

Niclosamide is widely used to control snails that transmit schistosomiasis. However, it can also harm non-target freshwater snails with important ecological functions. In this study, the mud snail *Cipangopaludina cathayensis* was exposed to environmentally relevant concentrations of niclosamide for 60 days to evaluate its chronic toxic effects. Niclosamide accumulated more in the digestive glands than in the foot, causing tubular atrophy, inflammatory responses, and depletion of essential nutrients. Structural damage was also observed in the foot tissue, including vacuolization and muscle fiber atrophy. Alteration in the nutrient metabolism in the digestive glands may adversely influence foot function, indicating a metabolic linkage between these organs. Such impairments could compromise snail survival, feeding, and movement, thereby posing potential ecosystem risks. These findings underscore the need for more environmentally responsible use of niclosamide.

## 1. Introduction

Niclosamide is the only molluscicide approved by the World Health Organization (WHO) for controlling *Oncomelania* snails in schistosomiasis [1], yet it poses notable risks to freshwater ecosystems. Field investigations have detected niclosamide residues in surface waters ranging from 0.13 to 38 µg/L [1,2,3], and its high chemical stability makes conventional drinking water treatment processes largely ineffective for its removal [4]. Niclosamide can accumulate in aquatic organisms and cause toxicity to non-target freshwater species, particularly mollusks [5,6]. Previous studies show that acute niclosamide exposure induces functional impairment of the digestive gland and foot in mollusks [7,8,9]. Mollusks play vital ecological roles and contribute substantially to freshwater food webs [10]. Evaluating the chronic toxicity of niclosamide is essential for accurately assessing ecological risks.

The digestive gland is central to nutrient metabolism and absorption [11] and plays a crucial role in stress responses in mollusks [7]. It is the primary site for the bioaccumulation and toxic effects of lipophilic pollutants [12]. These pollutants subsequently induce histopathological changes such as vacuolization, secretory cell necrosis, and degeneration of digestive tubules [13,14]. These pathological changes may compromise metabolic functions and lead to significant reductions in nutrient levels [15,16], thereby impairing the growth and survival of mollusks [17]. Previous research indicated that the digestive gland is the main organ responsible for mobilizing energy reserves [18].

The foot is continuously exposed to environmental pollutants, making it a direct target for pollutant toxicity [19]. Such exposure causes tissue damage [20,21] and impairs essential functions, including feeding and escape behaviors [22]. As the primary locomotory organ of mollusks, the foot has extremely high energy requirements and is highly sensitive to metabolic disturbances [23]. Evidence also suggests that the structural organization of the foot may be closely related to its energy storage capacity. Starvation induces atrophy of secretory glands in the foot, reduces its protein synthesis, and consequently weakens its attachment performance [24]. In addition to the energy stored within its own tissues, the foot also relies on energy supplied by the digestive gland [25]. In *Haliotis discus hannai* larvae, the digestive gland is involved in settlement by regulating glycogen breakdown and glycolytic energy supply [26].

We selected *C. cathayensis* as the study organism. It is a native species widely distributed in schistosomiasis-endemic regions of East Asia [27]. *C. cathayensis* is a key species in freshwater food webs and in water quality regulation, rendering it a representative indicator for evaluating the ecological risks of pollutants in regional aquatic ecosystems [28]. Notably, it does not serve as an intermediate host for *Schistosoma japonicum* [29]. Considering the propensity of niclosamide for lipophilic enrichment, we hypothesize that the digestive gland would serve as the primary site of accumulation during chronic exposure. Such accumulation is expected to induce structural injury in the digestive gland, thereby causing nutritional disturbances. We further hypothesize that impaired energy supply from the digestive gland may exacerbate structural damage in the foot. The aim of this study is to investigate the effects of a 60-day niclosamide exposure at environmentally relevant concentrations on *C. cathayensis* by (1) quantifying niclosamide bioaccumulation and changes in nutritional components in the digestive glands and foot; (2) characterizing histopathological lesions and inflammatory responses; and (3) exploring the toxic cascade between the digestive glands and foot. These findings will address the knowledge gap regarding nutrient-associated effects of chronic niclosamide exposure across multiple mollusks organs. They will also provide essential data for establishing environmental safety thresholds and support the science-based application of niclosamide.

## 2. Materials and Methods

### 2.1. Mesocosm Setup

**Figure 1:**
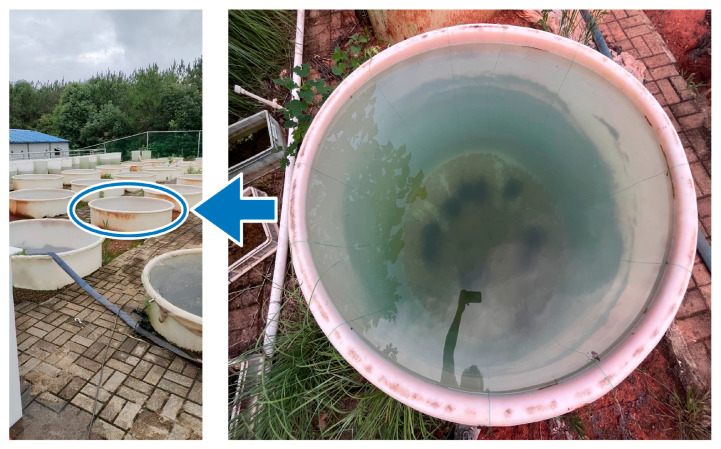
Photographs of the outdoor mesocosms used for chronic niclosamide exposure in *C. cathayensis*.

A 60-day outdoor mesocosm experiment was conducted at Hsen-Hsu Garden, Nanchang University, from late July to late September 2023. Twenty-five cylindrical PVC mesocosms (150 cm diameter × 160 cm depth; 2500 L) were established and filled with prefiltered pond water (100 μm mesh) from a nearby wetland (Figure 1). To simulate benthic conditions, 12 pots (22 cm diameter) contained 15 cm of homogenized Poyang Lake sediment. Prior to the start of the exposure period, mesocosms were hydraulically connected during a 30-day equilibration period, with one-third of the water exchanged weekly to homogeneous water quality. Initial TN and TP were 0.49 ± 0.11 mg/L and 0.02 ± 0.01 mg/L, respectively.

### 2.2. Niclosamide Exposures

A control and four niclosamide exposure levels (7.5, 15.0, 30.0, and 50.0 µg/L) were randomly assigned, with five mesocosms per treatment. Concentrations were chosen based on reported niclosamide residues (0.13–38 µg/L) in Poyang Lake and Nanjing surface waters [2,3], encompassing primarily environmentally relevant levels. The highest concentration (50 µg/L) corresponds to the surface water limit enforced in the United States [30].

Niclosamide (≥98%, Sigma-Aldrich, St. Louis, MO, USA) was administered every 48 h to compensate for photolytic degradation and sorption losses. Stock solutions were prepared in dimethyl sulfoxide (DMSO), and the final DMSO concentration was ≤0.05% (*v*/*v*) in all groups. Dosing was gently poured onto the water surface and immediately stirred with an 80 cm long stirring device for 30 s to ensure homogeneous mixing. To minimize concentration fluctuations caused by evaporation, dechlorinated tap water was added when water loss exceeded 5 cm.

Before each dosing event, water samples were collected 4–10 h prior and again 1 h after dosing. Samples were quantified using HPLC (Waters); detailed information is provided in the Supplementary Materials. Because mesocosm dosing created a non-steady-state exposure profile, time-weighted mean (TWM) concentrations were used for all analyses. TWM concentrations were 6.6 ± 1.31 µg/L (7.5 µg/L nominal), 11.76 ± 3.59 µg/L (15 µg/L), 26.20 ± 5.21 µg/L (30 µg/L), and 43.40 ± 7.81 µg/L (50 µg/L). The full temporal profile is shown in Figure S1.

### 2.3. C. cathayensis Collection and Analysis

*C. cathayensis* were purchased from Jiangxi Agricultural Development Group Co., Ltd. (Nanchang, China). Upon arrival, snails were acclimated for 14 days in aerated, dechlorinated water (22 ± 1 °C, 12 L/12 D). Individuals with uniform morphology (shell height 39.4 ± 0.4 mm, body mass 15.5 ± 1.5 g) were used for the experiment. Thirty snails were stocked into each mesocosm. Mortality was monitored daily and dead individuals were removed immediately. The mortality curves are presented in Figure S2, and the numbers of surviving *C. cathayensis* are listed in Table S1.

#### 2.3.1. Sampling Design

At the end of exposure, five surviving snails were randomly collected from each mesocosm. Half-sections of the digestive gland and foot were dissected and fixed; the remaining halves were pooled to form one combined sample per mesocosm. These composite samples were used for niclosamide bioaccumulation and nutritional component analyses. To ensure data reliability, biological replication was defined at the mesocosm level (*n* = 5 per treatment), and all niclosamide bioaccumulation and nutritional measurements were performed on mesocosm-level composite pools. *C. cathayensis* individuals were anesthetized by immersion in MS-222 solution (150 mg/L; Sigma-Aldrich, St. Louis, MO, USA). Mortality was defined as the absence of response to needle stimulation.

#### 2.3.2. Niclosamide Bioaccumulation Analysis

Tissue samples weighing 0.1 g were homogenized in acetonitrile (10 mL), sonicated (10 min), and centrifuged (10,000× *g*, 5 min, 4 °C). Supernatants (500 µL) were analyzed using the HPLC conditions described above. Bioconcentration factors (BCF) were interpreted as kinetic BCF [31].

#### 2.3.3. Nutritional Component Analysis

Combined samples of the digestive gland and foot were analyzed for protein (Kjeldahl method, conversion factor 6.25), lipid (chloroform–methanol extraction) [32], and glycogen (commercial kit, Nanjing Jiancheng, Nanjing, China, A048-2-1). All measurements were expressed per gram of wet weight.

#### 2.3.4. Histological Analysis

Foot and digestive gland tissues were fixed in 4% PFA for 72 h, dehydrated, and embedded in paraffin [7]. One slide was prepared from each snail for each tissue, five slides per mesocosm. Sections (5 µm) were stained with H&E and imaged on Olympus CX41 (Olympus, Tokyo, Japan). Digestive tubules phases (holding, adsorption, atrophy) were quantified according to Rodrigues et al. [33]. Hemocytic infiltration and nodules were quantified in 30 randomly selected fields per slide, with non-overlapping microscopic fields [33]. Morphometric parameters of the foot (fold depth, muscle fiber diameter, fiber density, vacuole density and area fraction) were measured using ImageJ (v.1.53a). To assess observer bias, histological scoring was independently performed by two trained observers.

### 2.4. Statistical Analysis

The kinetic BCF (L/kg) was determined using the following equation [34]:

(1) $$BCF=\frac{C_{T}}{C_{W}}$$

where C_T_ is niclosamide concentration (g/kg wet weight) and C_W_ is the TWM water concentration (g/L).

Since the data failed to pass the Shapiro–Wilk normality test, non-parametric tests were employed. Differences among treatments were evaluated using Kruskal–Wallis tests followed by Dunn’s post hoc tests with Benjamini–Hochberg FDR correction. Generalized additive models (GAMs) were used to quantify the concentration–response relationships between niclosamide exposure and each pathological index using the ‘mgcv’ package (v. 1.9-4) [35]. The effective degrees of freedom (EDF) of each smooth term were estimated to characterize the degree of nonlinearity. Models were fitted using penalized regression splines with a Gaussian error structure, and smoothing parameters were estimated by restricted maximum likelihood. Prior to GAM fitting, all indicators were normalized using min–max scaling (x′ = (x − min)/(max − min)) to standardize variables to a dimensionless range of 0–1. The raw value ranges for each index are provided in Table S4. Relationships between niclosamide concentrations, nutritional endpoints, and histopathological indices were analyzed using Spearman rank correlations implemented with the ‘stats’ package [36]. All analyses were performed in R (v.4.3.1).

## 3. Results

### 3.1. Niclosamide Bioaccumulation and Nutrient Content Changes

**Figure 2:**
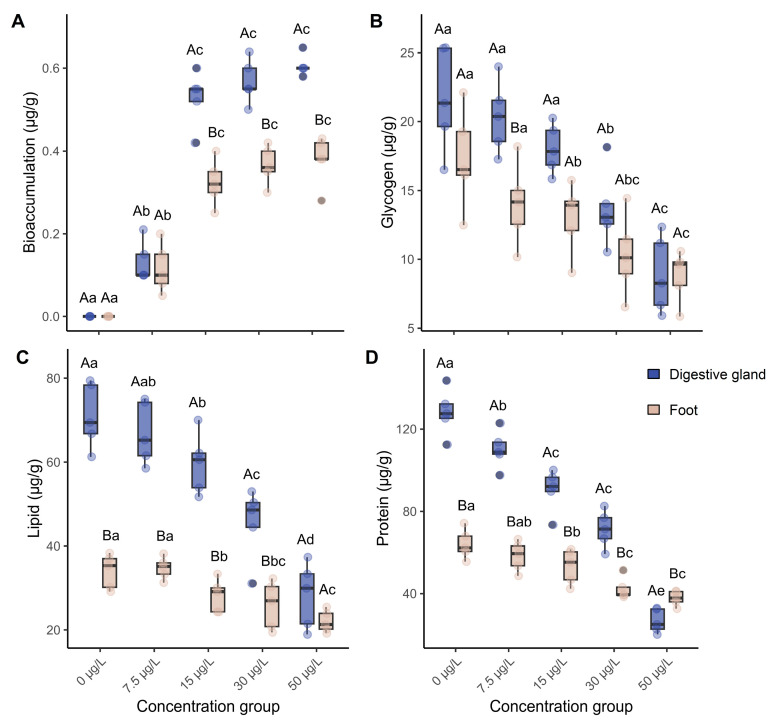
Niclosamide bioaccumulation and nutrient contents in the digestive glands and foot tissues of *C. cathayensis* following a 60-day exposure. (**A**) Niclosamide bioaccumulation, (**B**) glycogen, (**C**) lipid, (**D**) protein. Capital letters indicate significant differences (*p* < 0.05) between tissues at the same exposure concentrations, whereas lowercase letters indicate significant differences (*p* < 0.05) between concentrations within the same tissue. Data are presented as mean ± SD (*n* = 5).

Following a 60-day exposure, niclosamide accumulated in both the digestive glands and the foot of *C. cathayensis*. Accumulation was tissue-specific, as digestive glands contained significantly higher niclosamide levels than foot tissues at concentrations ≥15 µg/L (*p* < 0.05; Figure 2A). Dose-dependent accumulation was observed, with concentrations reaching 0.61 ± 0.02 µg/g in digestive glands and 0.38 ± 0.02 µg/g in foot tissues at 50 µg/L exposure. Kinetic BCF ranged from 12.1 to 35.2 L/kg in digestive glands and from 7.6 to 21.6 L/kg in foot tissues across all treatments, with coefficients of variation ranging from 12.8 to 51.2% and 7.1 to 36.9%, respectively (Table S2).

Glycogen, lipid, and protein contents decreased across all niclosamide exposure groups (Figure 2B–D). At 50 µg/L, lipid levels in the digestive gland dropped by 60.6%, glycogen levels by 59.3%, and protein levels by 79.2% relative to the control group.

### 3.2. Structural and Functional Impairment of Digestive Glands

**Figure 3:**
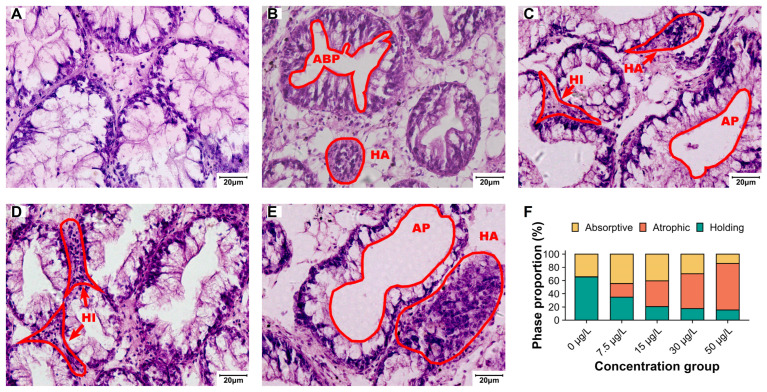
Histopathology of digestive glands (H&E, ×200) and distribution of digestive tubule phases following a 60-day exposure. (**A**) Control group, (**B**) 7.5 µg/L group, (**C**) 15 µg/L group, (**D**) 30 µg/L group, (**E**) 50 µg/L group, and (**F**) distribution of digestive tubule phases. ABP denotes absorptive phase tubules and AP denotes atrophic phase tubules. Hemocyte infiltration (HI) and hemocyte nodules (HA) are also indicated. Scale bar = 20 µm. Data are presented as mean (*n* = 5).

Control digestive glands showed intact tubular structures with connective tissue and hemolymph sinuses (Figure 3). Histopathological analysis classified tubules into three phases, the holding phase, the absorptive phase, and the atrophic phase. The specific characteristics of the three phases are shown in Table S3.

**Figure 4:**
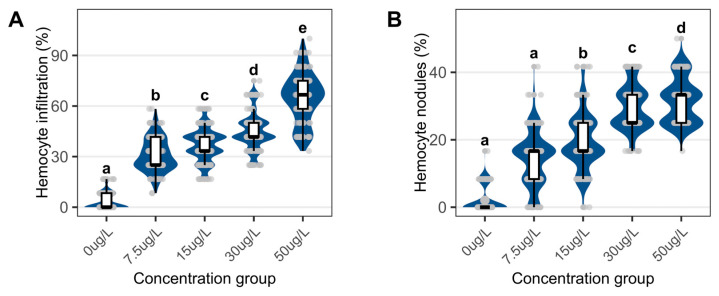
Quantitative assessment of digestive gland inflammatory responses in *C. cathayensis* following a 60-day exposure. (**A**) Proportion of hemocyte infiltration and (**B**) proportion of hemocyte nodules. Lowercase letters indicate significant differences (*p* < 0.05) between concentrations. Data are presented as mean ± SD (*n* = 5).

Niclosamide treatment reduced the proportion of the holding phases’ digestive tubules while increasing atrophic phases tubules (Figure 3F) and exhibited elevated hemocyte infiltration and nodular formation (Figure 4).

### 3.3. Structural Changes in Foot

**Figure 5:**
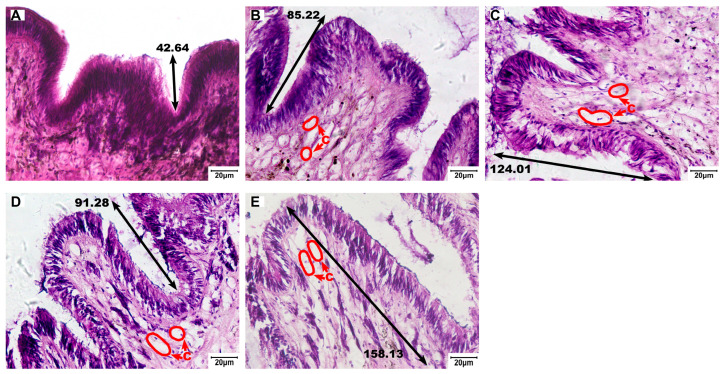
Epidermal histopathology in the foot of *C. cathayensis* following a 60-day exposure (H&E, ×200). (**A**) Control group, (**B**) 7.5 µg/L group, (**C**) 15 µg/L group, (**D**) 30 µg/L group, and (**E**) 50 µg/L group. Vacuolation is denoted by C. Fold depths are labeled with numbers. Scale bar = 20 µm.

**Figure 6:**
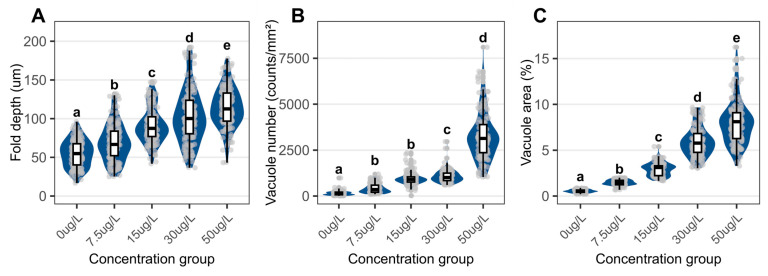
Quantitative assessment of epidermal parameters in the foot of *C. cathayensis* following a 60-day exposure. (**A**) Fold depth, (**B**) vacuole numerical density, and (**C**) vacuolar area fraction. Lowercase letters indicate significant differences (*p* < 0.05) between concentrations. Data are presented as mean ± SD (*n* = 5).

**Figure 7:**
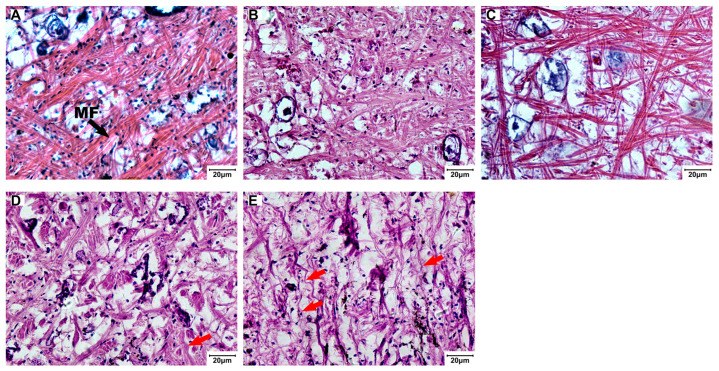
Muscle tissue histology of the foot in *C. cathayensis* following a 60-day exposure (H&E, ×200). (**A**) Control group, (**B**) 7.5 µg/L group, (**C**) 15 µg/L group, (**D**) 30 µg/L group, and (**E**) 50 µg/L group. Muscle fibers are labeled as MF. Red arrows indicate curved fibers. Scale bar = 20 µm.

The foot tissues of control snails exhibited densely arranged epithelial and muscular layers with shallow, smooth epithelial folds (Figure 5A, Figure 6 and Figure 7A). Niclosamide treatment increased epithelial fold depth from 54.99 ± 17.36 µm in control to 114.45 ± 25.85 µm at 50 µg/L (Figure 6A). Vacuolation was evident in exposed groups, with the vacuolar area fraction rising from 0.53 ± 0.17% (control) to 7.99 ± 2.39% (50 µg/L; Figure 6C).

**Figure 8:**
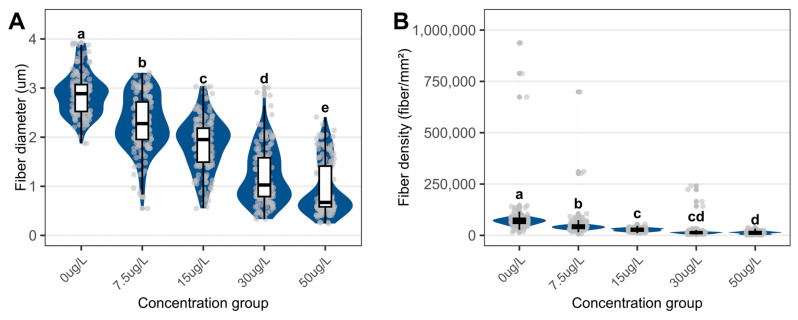
Quantitative assessment of myofiber parameters in the foot of *C. cathayensis* following a 60-day exposure. (**A**) Muscle fiber diameter and (**B**) muscle fiber density. Lowercase letters indicate significant differences (*p* < 0.05) between concentrations. Data are presented as mean ± SD (*n* = 5).

Muscle fiber diameter decreased from 2.88 ± 0.44 µm (control) to 0.96 ± 0.55 µm (50 µg/L), and fiber density dropped from 86,244 ± 10,539 to 13,602 ± 7223 fibers/mm^2^ (Figure 8).

### 3.4. Relationships Between Niclosamide Exposure, Bioaccumulation, Nutrient Components, and Histopathology

**Figure 9:**
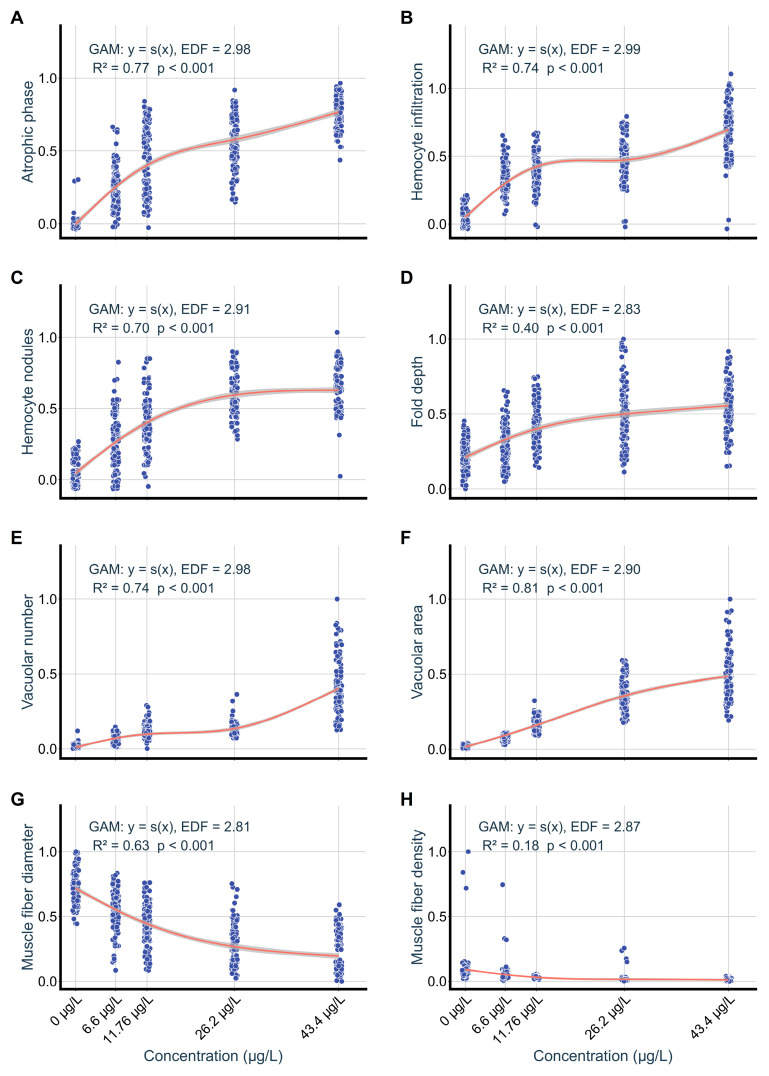
GAMs describing concentration–response for normalized pathological indices in *C. cathayensis* following a 60-day exposure. Digestive gland parameters (**A**) atrophic phase proportion, (**B**) hemocyte infiltration, (**C**) hemocyte nodules. Foot parameters, (**D**) fold depth, (**E**) vacuolar numerical density, (**F**) vacuolar area fraction, (**G**) muscle fiber diameter, (**H**) muscle fiber density. The red solid lines represent the fitted GAM curves, and the blue circles indicate the observed values. EDF (Effective Degrees of Freedom) is provided for each model to indicate the degree of non-linearity.

Normalized pathological indices showed concentration-dependent trends (Figure 9). Vacuolar area in foot tissues was correlated with exposure concentrations (R^2^ = 0.81, *p* < 0.001), whereas muscle fiber density showed a weaker association (R^2^ = 0.17, *p* < 0.001). Digestive gland lesions increased with exposure levels, particularly atrophied tubules (R^2^ = 0.77, *p* < 0.001) and hemocyte infiltration (R^2^ = 0.74, *p* < 0.001).

**Figure 10:**
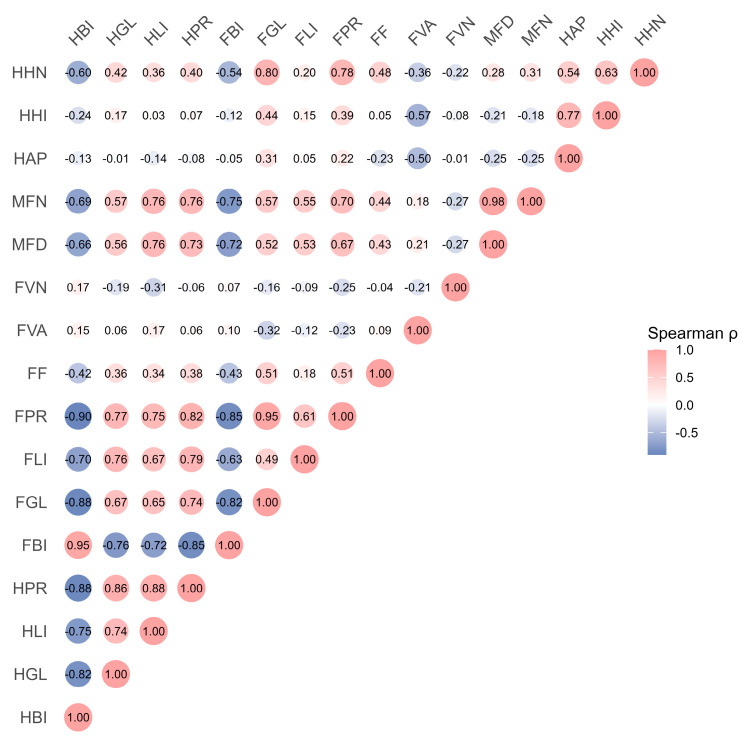
Spearman rank correlations among niclosamide bioaccumulation, nutrient components, and histopathological parameters in digestive glands and foot tissues. Digestive gland indices HBI (niclosamide), HGL (glycogen), HLI (lipid), HPR (protein), HAP (atrophic phase proportion), HHI (hemocyte infiltration), and HHN (hemocyte nodules). Foot indices FBI (niclosamide), FGL (glycogen), FLI (lipid), FPR (protein), FF (fold depth), FVN (vacuolar numerical density), FVA (vacuolar area fraction), MFD (muscle fiber diameter), MFN (and muscle fiber density). The size of the circles represents the absolute value of the correlation coefficient.

Spearman correlation analysis indicated that nutrient contents of digestive glands were positively associated with foot tissue nutrient levels, especially for protein (r = 0.82, *p* < 0.05; Figure 10). Foot tissue nutrient levels also correlated positively with muscle fiber indices (r = 0.43–0.70, all *p* < 0.05). Meanwhile, niclosamide concentrations were negatively correlated with both nutrient concentrations and muscle fiber diameter (r ≤ −0.63, *p* < 0.05; r ≤ –0.72, *p* < 0.001, respectively).

## 4. Discussion

Widespread use of niclosamide led to residues in surface waters and induced toxicity in aquatic organisms, raising substantial ecological concern [5,37]. In this study, we integrated biochemical indicators with histopathological alterations in the mud snail (*C. cathayensis*) under environmentally relevant exposure levels. Our objective was to determine how niclosamide exposure affects the cross-organ associations of nutrition-related parameters.

### 4.1. Bioaccumulation of Niclosamide

In this study, we demonstrate that the digestive glands of *C. cathayensis* serve as the primary reservoir for niclosamide accumulation. Niclosamide is lipophilic [38] and may passively diffuse across mollusk epidermal barriers, facilitating intestinal uptake and preferential accumulation in lipid-rich tissues such as digestive glands [6,39].

The kinetic BCF in the digestive glands of *C. cathayensis* was higher than values reported for amphibians but slightly lower than those for fish [40,41]. These differences likely reflect species-specific exposure duration and metabolic capacities, as amphibians in Xiang et al.’s study could temporarily avoid contaminated habitats [40].

### 4.2. Structural and Functional Impairment of Digestive Glands Induced by Niclosamide

Our findings reveal that niclosamide exposure induces severe pathological alterations in the digestive glands of *C. cathayensis*, including atrophy of digestive tubules, hemocyte infiltration, and nodular formations. The salicylanilide structure of niclosamide has been reported to enable hydrogen-bond interactions with nitric oxide synthase (NOS) in other species [42], which may suppress NOS activity and promote hemocyte-mediated inflammatory responses [7,43,44]. Previous studies on *Pomacea canaliculate* have reported similar histopathology under acute niclosamide exposure [7].

Reduced nutrient levels (Figure 1) suggest that atrophy phase tubules may impair nutrient digestion and absorption efficiency [33]. In summary, the results point to structural degeneration and possible functional impairment of the digestive gland in *C. cathayensis*.

### 4.3. Structural and Metabolic Pathologies in the Foot Induced by Niclosamide

Vacuolar expansion in the foot likely reflects hypersecretory responses that disrupt intracellular metabolic homeostasis [45], a protective reaction reported in mollusks exposed to pollutants [46]. Similar vacuolation has been observed in *Monacha cartusiana* after exposure to *Bacillus thuringiensis* [47]. The positive association between niclosamide concentration and the vacuolar area in foot tissue (R^2^ = 0.81, *p* < 0.001) could further support the role of hypersecretion.

Spearman correlations showed (1) positive associations between nutrient levels in the digestive gland and foot, (2) negative associations between foot nutrients and muscle fiber diameter, and (3) negative associations of niclosamide exposure with nutrient levels and muscle fiber diameter. These results suggest a nutritional linkage between the digestive glands and foot, indicating that the digestive glands may act as a primary nutrient source. Because the digestive gland is central to nutrient metabolism and absorption [11], its impairment might reduce nutrient supply to dependent tissues such as the foot [48]. The high energy demand of the foot increases its vulnerability to such metabolic shortfalls [23]. Evidence indicates that biopesticide-induced digestive gland impairment restricts nutrient supply to muscular tissues [47]. Niclosamide exposure induced energy-metabolism disorders in the digestive gland of *Pomacea canaliculata* and disrupted transmembrane transport processes in the foot [7]. These observations suggest a potential metabolic linkage between the two organs that may be disrupted by niclosamide. Further enzymatic, transcriptomic, and hemolymph analyses are required to verify this mechanism in *C. cathayensis*.

### 4.4. Ecological Impacts of Niclosamide Exposure

Niclosamide exposure caused structural damage and functional impairment in the digestive glands and foot muscles of *C. cathayensis*, which may compromise survival, growth, and reproduction [49]. In this study, we observed snail mortality under experimental exposure (Table S1), indicating that chronic or repeated exposures could destabilize local mollusk populations [4]. Beyond mollusks, niclosamide has demonstrated broad-spectrum ecotoxicity, inducing growth inhibition and acute lethality in fish, crustaceans, and amphibians [38,40]. Prolonged contamination may thus impair ecosystem resilience and disrupt processes such as nutrient cycling and energy flow [38,50].

As the primary molluscicide for schistosomiasis control in many regions of Asia, Latin America, and Africa [19,51], niclosamide use requires balancing public health benefits with environmental protection. Field surveys have reported frequent niclosamide detections in surface waters of the Yangtze River Basin (95% of samples) [3]. Most nations currently lack enforceable regulations to control emissions, while only the United States enforces a surface water limit of 50 µg/L [30]. Improved knowledge of niclosamide toxicity can guide the development of ecologically sound safety standards and more effective vector-control measures.

## 5. Conclusions

This study evaluated niclosamide bioaccumulation and its chronic effects in *C. cathayensis*, with a focus on histopathological alterations and changes in tissue nutrient composition. After 60 days of exposure, niclosamide accumulated in both the foot and the digestive glands. Significant histopathological damage was induced in these organs, accompanied by reduced glycogen, lipid, and protein concentrations. The observed correspondence between digestive gland nutrient reduction and foot tissue damage suggests a disruption of metabolic linkage between these organs. Together, these findings provide organ-level evidence that prolonged niclosamide exposure poses potential risks to non-target freshwater mollusks and offers important data for ecological risk assessment. Further studies incorporating additional species and mechanistic endpoints are needed to better evaluate the broader ecological implications of long-term niclosamide use.

## Data Availability

All original data and materials presented in this study are included in the article and Supplementary Materials. Further inquiries can be directed to the corresponding authors.

## Supplementary Materials

The following supporting information can be downloaded at: https://www.mdpi.com/article/10.3390/biology15010102/s1, Figure S1: Temporal trends of measured NIC concentrations for mesocosm water across all treatment groups during the experimental period; Figure S2: Mortality curves of *C. cathayensis* following a 60-day niclosamide exposure; Table S1: Number of surviving *C. cathayensis* per PVC mesocosm following a 60-day niclosamide exposure; Table S2: Mean, SD, and CV of kinetic BCF in digestive gland and foot; Table S3: Histological characteristics of functional phases in digestive tissues; Table S4: Original data ranges for pathological indices before normalization.
