# Supplementary material for: Chronic Exposure to Niclosamide Disrupts Structure and Metabolism of Digestive Glands and Foot in Cipangopaludina cathayensis
Source: Biology (Basel). 2026 Jan 4;15(1):102. doi: 10.3390/biology15010102 (PMC12785012; doi:10.3390/biology15010102)
Supplement: Supplementary file 1 [file biology-15-00102-s001.zip › biology-4031155-Supplementary material.pdf]

Supplementary Information

Chronic Exposure to Niclosamide Disrupts Structure and Metabolism of Digestive Glands and Foot in *Cipangopaludina cathayensis*

Yanan Zhang[1], Yizhen Liu[1,2]*, Qiying Cai[1], Jun Ye[1], Tao Wang[1], Sheng Xu[1], Gang Ge[1,2]*

[1] School of Life Science, Key Laboratory of Poyang Lake Environment and Resource Utilization, Ministry of Education, Nanchang University, Nanchang 330031, China;
[2] Jiangxi Provincial Key Laboratory of Poyang Basin Ecological Hydrological Monitoring, Nanchang 330031, China

* Correspondence: liuyizhen@ncu.edu.cn (Y.L.); gge@ncu.edu.cn (G.G.)

Summary: Supporting Information provides a summary of experimental details and data, including: (i) method for the determination of niclosamide content; (ii) calculation methods for FVN, FVA, and MFN; (iii) water concentrations of niclosamide during the experiment; (iv) mortality curves of *C. cathayensis*; (v) the numbers of surviving *C. cathayensis* following 60-day exposure, (vi) mean, SD, and CV of kinetic BCF in digestive gland and foot; (vii) histological characteristics of functional phases in digestive tissues; and (ix) original data ranges for pathological indices before normalization.

1. Supplementary Methods

The concentration of niclosamide was determined as follows: Samples were kept in amber vials, frozen (−20°C), dried at 45°C under nitrogen, reconstituted (acetonitrile/water 1:10), purified via C18 SPE, and filtered (0.22-µm). Niclosamide was quantified using HPLC (Waters C18 column, 250 × 4.6 mm, 5 µm; 25°C; 1.0 mL/min; 236 nm detection). Limit of detection (LOD) and limit of quantification (LOQ) were 1 and 3 ng/mL. Values <LOD were categorized as zero.

Vacuole numerical density (FVN) was defined as:

$$\mathbf{FVN} = \frac{\mathbf{N_V}}{\mathbf{A}}$$

where Nv is the number of vacuoles within the measurement area and A is the standardized sampling area (mm²), expressed as counts/mm².

Vacuole area fraction (FVA) was calculated as:

$$\mathbf{FVA} = \frac{\mathbf{A_v}}{\mathbf{A}} \times \mathbf{100\%}$$

where Av is the cumulative vacuole area (mm²) and A is the sampling area (mm²).

Muscle fiber density (MFN) was determined as:

$$\mathbf{MFN} = \frac{\mathbf{N_m}}{\mathbf{A}}$$

where Nm is muscle fiber count and A is the sampling area (mm²), with units of fibers/mm².

2. Supplementary Figures

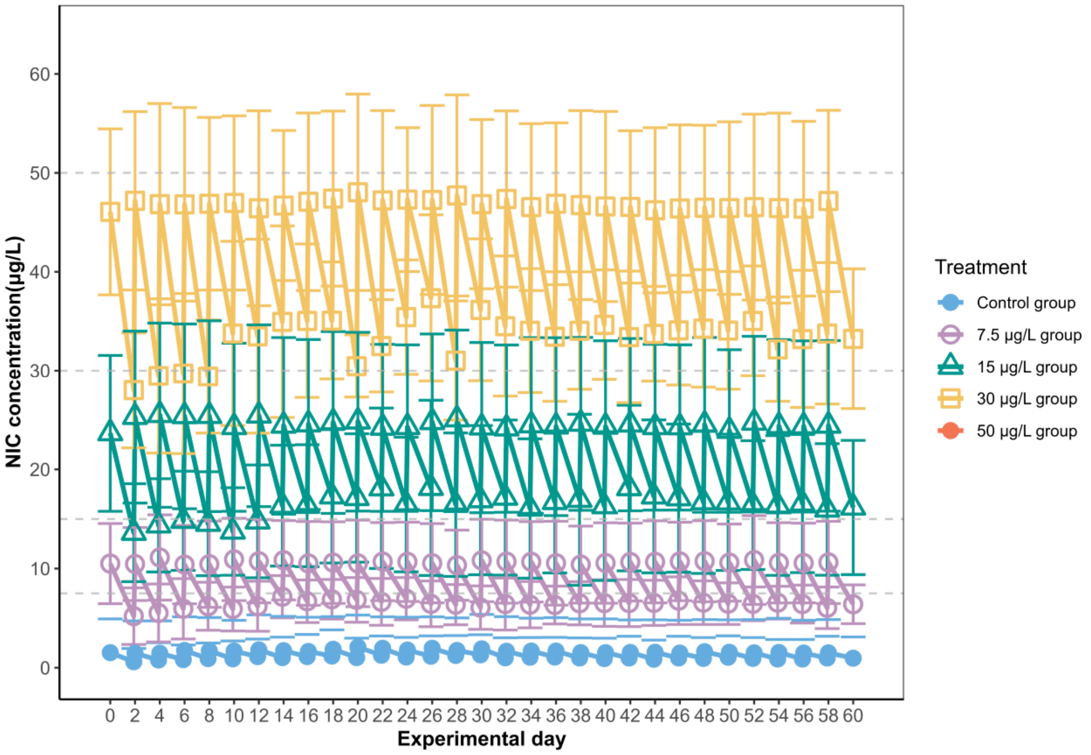

Figure S1. Temporal trends of measured niclosamide concentrations for mesocosm water across all treatment groups during the experimental period. Data are presented as means ± SD (n = 5).

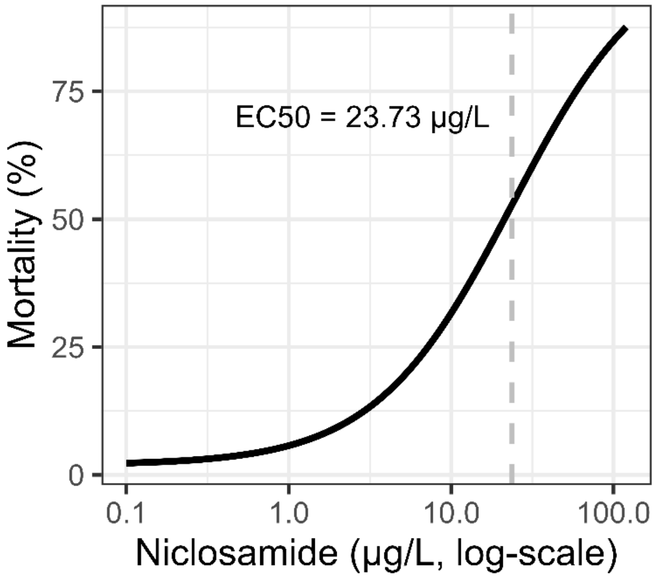

Figure S2. Mortality curves of *C. cathayensis* following a 60-day niclosamide exposure.

3. Supplementary Tables

Table S1. Number of surviving *C. cathayensis* per PVC mesocosm following a 60-day niclosamide exposure.

Treatment group	Mesocosm ID	Initial N (Day 0)	Survivors (Day 60)	% Survivors (Day 60)
Control	T0-1	30	30	100.00
Control	T0-2	30	30	100.00
Control	T0-3	30	29	96.67
Control	T0-4	30	29	96.67
Control	T0-5	30	29	96.67
7.5 µg/L	T1-1	30	21	70.00
7.5 µg/L	T1-2	30	20	66.67
7.5 µg/L	T1-3	30	24	80.00
7.5 µg/L	T1-4	30	23	76.67
7.5 µg/L	T1-5	30	23	76.67
15 µg/L	T2-1	30	18	60.00
15 µg/L	T2-2	30	16	53.33
15 µg/L	T2-3	30	17	56.67
15 µg/L	T2-4	30	20	66.67
15 µg/L	T2-5	30	18	60.00
30 µg/L	T3-1	30	12	40.00
30 µg/L	T3-2	30	13	43.33
30 µg/L	T3-3	30	15	50.00
30 µg/L	T3-4	30	12	40.00
30 µg/L	T3-5	30	9	30.00
50 µg/L	T4-1	50	12	40.00

50 μg/L	T4-2	50	10	33.33
50 μg/L	T4-3	50	6	20.00
50 μg/L	T4-4	50	7	23.33
50 μg/L	T4-5	50	8	26.67

Table S2. Mean, SD, and CV of kinetic BCF in digestive gland and foot (n=5).

Organ	Treatment Group (μg/L)	Mean	SD	CV (%)
Digestive gland	0	0.0	0.00	-
Digestive gland	7.5	17.6	5.30	36.9
Digestive gland	15	35.2	3.64	12.7
Digestive gland	30	18.9	1.46	9.4
Digestive gland	50	12.1	0.43	4.3
Foot	0	0.0	0.00	-
Foot	7.5	15.5	6.47	51.2
Foot	15	21.6	3.05	17.3
Foot	30	12.2	1.27	12.8
Foot	50	7.56	0.97	15.7

Table S3. Histological characteristics of functional phases in digestive tissues.

Phase	Characteristic histological changes
Holding	plump digestive cells
absorptive	irregular luminal contours with hypertrophied epithelial layers
atrophic	luminal dilation accompanied by cellular degeneration

Table S4. Original data ranges for pathological indices before normalization.

Parameter	Raw min	Raw max	Unit
Atrophic phase proportion	0	91.67	%
Hemocyte infiltration proportion	0	100.00	%
Hemocyte nodule proportion	0	50	%
Fold depth	16.66	192.30	μm
Vacuole numerical density	10.47	8103.19	counts/mm²
Vacuolar area fraction	0.20	16.24	%
Muscle fiber diameter	0.24	3.92	μm
Muscle fiber density	2221.07	937610.12	fibers/mm²
