# Supplementary figures and images for: Chronic Exposure to Niclosamide Disrupts Structure and Metabolism of Digestive Glands and Foot in Cipangopaludina cathayensis
Source: Biology (Basel). 2026 Jan 4;15(1):102. doi: 10.3390/biology15010102 (PMC12785012; doi:10.3390/biology15010102)

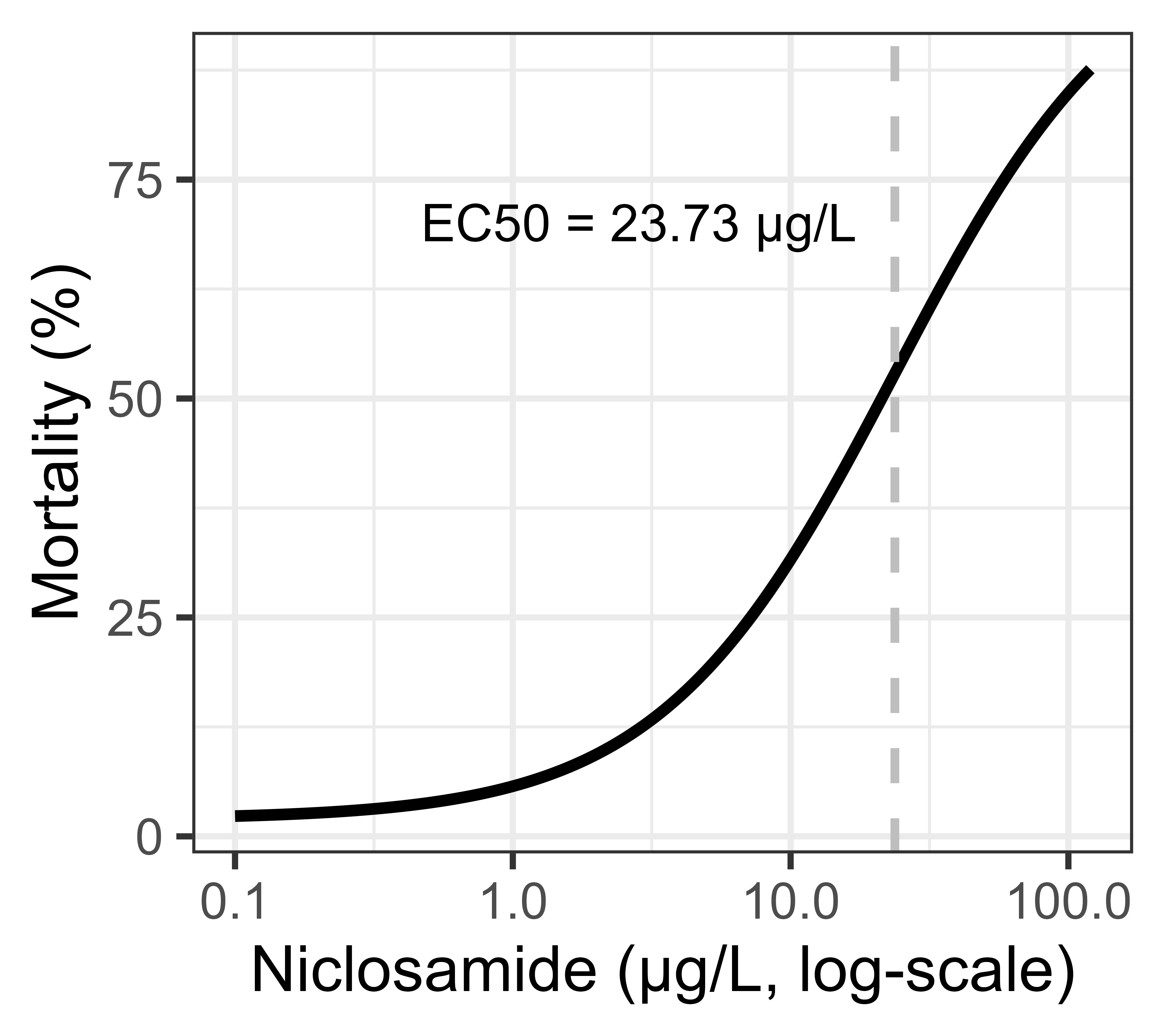

Supplement: Supplementary file 1 [file biology-15-00102-s001.zip › Figure_S2.png]
